# The Preliminary Education of Medical Students

**Published:** 1904-12-10

**Authors:** 


### THE PRELIMINARY EDUCATION OF MEDICAL STUDENTS.

It is many years since the preliminary education of medical students has attracted so much attention as it is doing at the present time. From the days of Queen Elizabeth onwards medical students have always been submitted to some test of knowledge before they were allowed to enter upon their purely professional education. Thus, in 1556, it is ordained amongst other articles of the United Company of Barbers and Surgeons that "no barbour surgeon that doth occupy the mystery of surgery shall take or have any apprentice but that he can skill of the Latin tongue and understand the same and can write and read sufficiently." In later years, when the Society of Apothecaries took charge of medical education under the Act of 1815, the preliminary examination was made a real test of the school education. The examination was conducted by the two wardens of the society who took passages from a book called "Selectæ profanis." The wardens retired from the table, each taking a candidate, and dealt faithfully with him in another part of the room for a quarter of an hour, twenty minutes, or perhaps half an hour. He then reported to the master his satisfaction or dissatisfaction with the youth's attainment, and the apprenticeship was either proceeded with or delayed upon the report. The examination thus conducted soon became a favourite one; it was sufficient, but not too difficult, and as the event proved was a test of good school teaching, for many of the medical men in the last century were better classical scholars than those who are now being educated. The Royal College of Surgeons of England usually accepted the preliminary examination of the Society of Apothecaries as a test of the general education of their members, but when the higher grade of a Fellow was established in 1843 a somewhat more searching preliminary examination was exacted. This, however, has fallen into abeyance for many years past. Of late years the basis of preliminary education has been greatly widened, and what has been gained in breadth has been lost in depth. Recent discussions have shown that in the minds of many it is desirable to curtail the number of subjects required of an average boy at the age of 16–19, who has received the education given at an ordinary English school. Just now the battle rages round the teaching of Greek, and does not immediately concern the medical student who has long been able to obtain the highest medical and surgical degrees without a knowledge of even the rudiments of this language. But incidentally the whole subject of preliminary education is raised and there is no question that our preliminary examination is insufficient as a test of general knowledge, and that the average student now is not so well prepared for his professional work as he was twenty years ago when fewer subjects were required of him. He is still most deficient in those subjects which ought to come most naturally, spelling and English composition; his mathematics are better, but his Latin is beneath contempt and he has no Greek. Fortunately, however, he is still in *statu pupillari*—many written examinations in class, and at the Examination Hall, the writing of daily ward-notes, and the general development of his knowledge gradually improve him, so that by the time he is 21 and qualified, he can occasionally send to the medical periodicals a decently-worded record of an interesting case which has come under his observation. There is no doubt, however, that the time is at hand when the schools must remodel their course for those who intend to take up medicine or science as a career; less time must be devoted to grammar and more time to chemistry and physics. Some schools, indeed, have already taken this important step, but it is obvious from the pupils who come to the hospitals that much more remains to be done if we are to get the best results.

